# Supplementary material for: Archaean and Proterozoic diamond growth from contrasting styles of large-scale magmatism
Source: Nat Commun. 2017 Sep 21;8:648. doi: 10.1038/s41467-017-00564-x (PMC5608721; doi:10.1038/s41467-017-00564-x)
Supplement: Supplementary file 1 — Supplementary Information [file 41467_2017_564_MOESM1_ESM.pdf]

Description of Supplementary Files

File Name: Supplementary Information
Description: Supplementary Figures, Supplementary Tables and Supplementary References

File Name: Supplementary Data 1
Description: Garnet inclusions major and trace element contents

File Name: Peer Review File

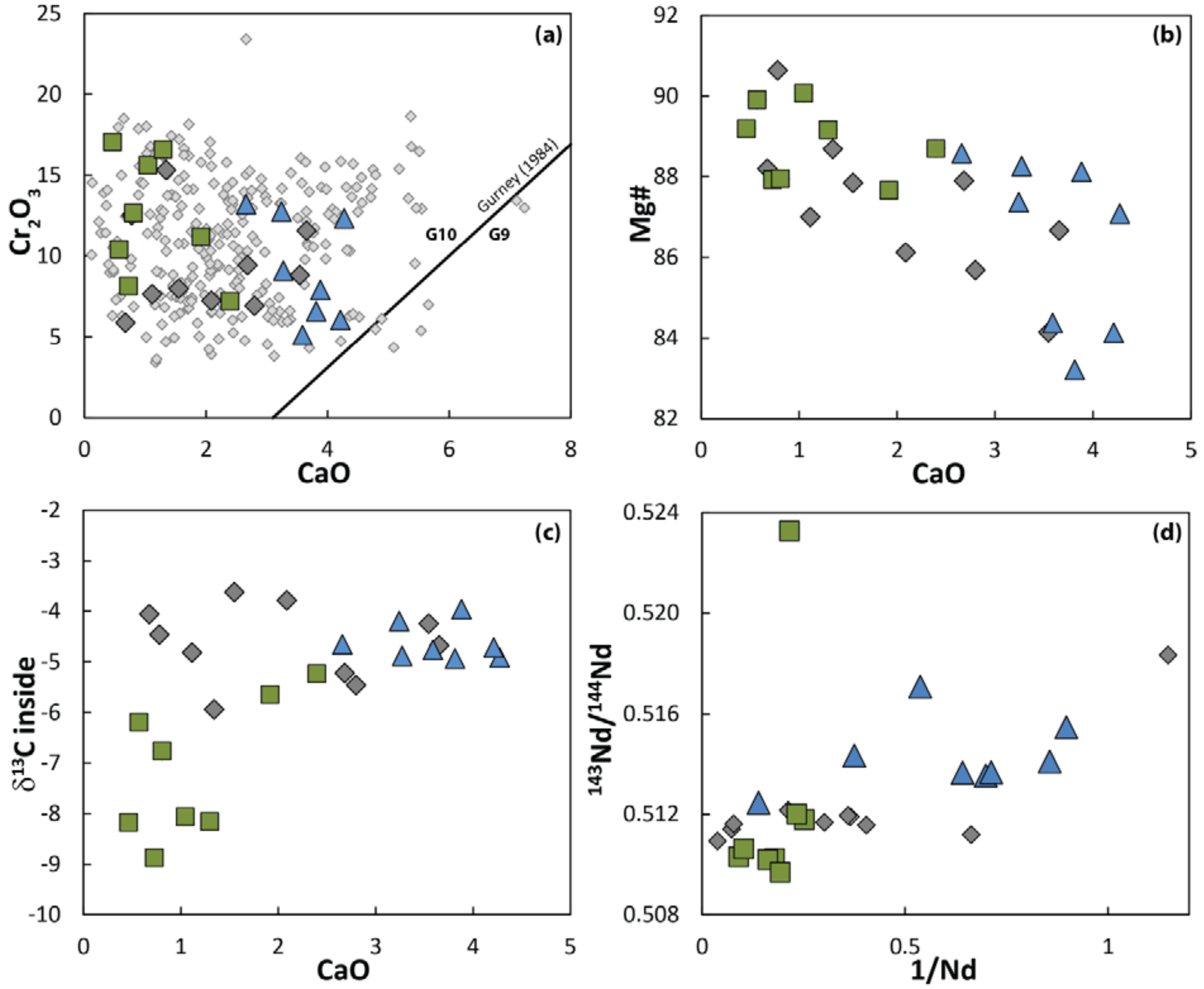

Supplementary Figure 1

Plots of Cr_2O_3, #Mg and carbon isotopes versus CaO for all garnet inclusions (a), (b) and (c) and between Nd isotope compositions and reciprocal Nd, (d). Symbols as in Figure 2 in main text. Small light grey diamonds in (a) are garnet inclusion data from Venetia by Richardson et al., 2009[1].

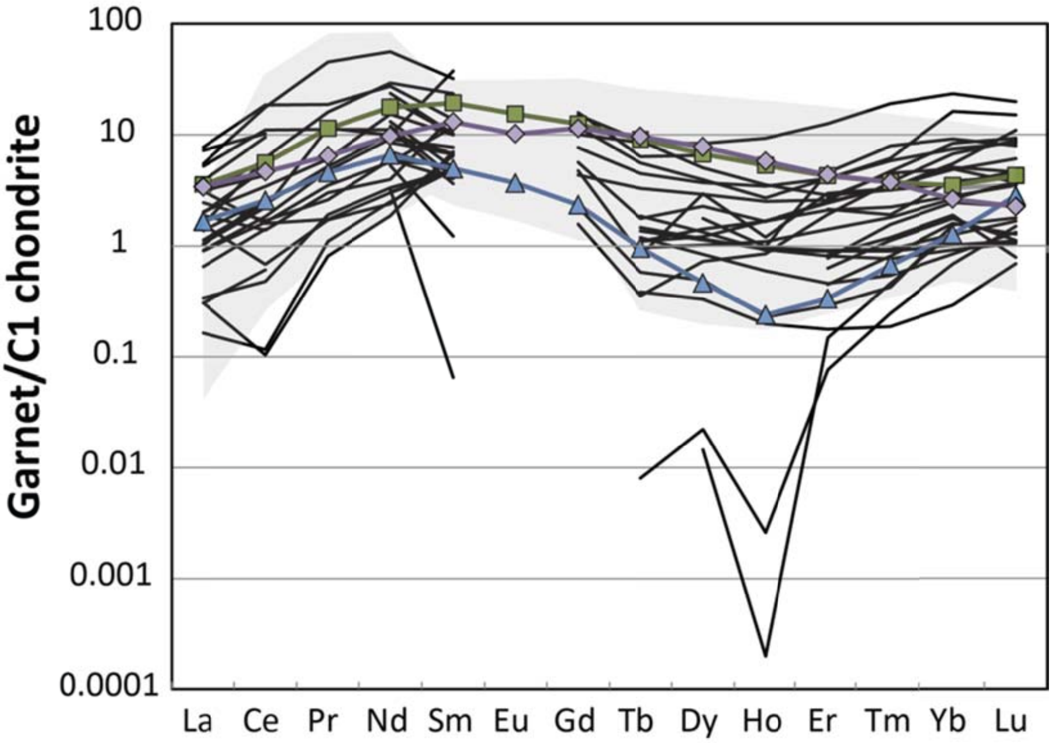

Supplementary Figure 2

Chondrite normalised rare earth patterns for all garnet inclusions including three inclusions (colored lines with symbols) that were not dated (see methods). The grey field represents SIMS data for garnet inclusions from Stachel et al., 2004[2]. Note that Sm and Nd concentrations for the inclusions processed through chemistry, are determined by ID-TIMS (Supplementary Table 2).

Supplementary Table 1

Carbon isotopes in host diamonds

Sample	Age Group*	$\delta^{13}C$ inside†	$\delta^{13}C$ rim
**V306**	-	-4.057	-4.378
**V327**	-	-5.219	-5.476
**V379**	3.0	-6.191	-5.115
**V405**	3.0	-8.064	-7.393
**V408**	3.0	-5.234	-5.026
**V445**	1.1	n.a.	-3.960
**V471**	-	-3.620	-3.097
**V488**	3.0	-8.872	-8.802
**V491**	-	-3.785	-3.858
**V517**	3.0	-8.158	-8.268
**V526**	-	-5.455	-5.077
**V536**	-	-4.459	-4.924
**V556**	1.1	-4.908	-5.149
**V569**	1.1	-4.881	-3.986
**V576**	-	-5.935	-5.479
**V583**	1.1	-4.713	-4.310
**V592**	3.0	-6.767	-5.420
**V601**	3.0	-5.647	-5.507
**V617**	-	-4.244	-4.224
**V641**	1.1	-4.191	-4.149
**V662**	1.1	-4.649	-4.374
**V663**	-	-4.812	-4.914
**V670**	-	-4.672	-4.722
**V674**	1.1	-4.931	-4.515
**V679**	1.1	-4.759	-4.571
**V683**	3.0	-8.178	-4.548

* Age group as in Table S1

†Inside fragments were picked fragments without crystal faces.

Supplementary Table 2

Sm-Nd concentrations and isotope compositions

Sample	Age group	weight mg	Nd ppm	Sm ppm	$^{147}Sm/^{144}Nd$	$^{143}Nd/^{144}Nd_m$	2SE	$^{143}Nd/^{144}Nd_e$	TDM Ga
**V306**	-	0.145	1.508	0.0099	0.004	0.51119	0.00007	0.51118	1.34
**V327**	-	0.212	2.736	0.8053	0.178	0.51191	0.00004	0.51130	4.14
**V379**	3.0	0.128	5.594	0.6349	0.069	0.51024	0.00004	0.51001	2.84
**V405**	3.0	0.089	11.059	1.5680	0.086	0.51031	0.00004	0.51002	3.11
**V408**	3.0	0.070	3.961	1.0270	0.157	0.51179	0.00006	0.51125	3.08
**V445**	1.1	0.230	1.430	0.7217	0.305	0.51353	0.00005	0.51249	0.76
**V471**	-	0.182	2.470	0.1871	0.046	0.51157	0.00005	0.51141	1.34
**V488**	3.0	0.061	6.162	0.7752	0.076	0.51020	0.00007	0.50994	3.02
**V491**	-	0.119	3.317	0.7077	0.129	0.51168	0.00006	0.51124	2.34
**V517**	3.0	0.054	9.701	1.5664	0.098	0.51063	0.00004	0.51030	3.02
**V526**	-	0.023	26.223	4.9179	0.113	0.51094	0.00003	0.51056	3.02
**V536**	-	0.052	13.693	3.6049	0.159	0.51141	0.00004	0.51086	4.10
**V556**	1.1	0.052	2.663	1.7908	0.407	0.51433	0.00007	0.51294	1.00
**V569**	1.1	0.026	1.114	0.9933	0.540	0.51547	0.00016	0.51363	1.13
**V576**	-	0.046	12.810	2.1084	0.100	0.51162	0.00007	0.51128	1.85
**V583**	1.1	0.055	1.167	0.7409	0.384	0.51409	0.00010	0.51278	0.92
**V592**	3.0	0.120	4.637	5.7519	0.752	0.52326	0.00005	0.52070	2.90
**V601**	3.0	0.088	4.274	1.1817	0.167	0.51201	0.00005	0.51144	3.05
**V617**	-	0.049	2.778	0.6191	0.135	0.51195	0.00015	0.51149	2.03
**V641**	1.1	0.082	7.164	1.5328	0.129	0.51246	0.00005	0.51202	1.07
**V662**	1.1	0.019	1.859	2.2720	0.740	0.51708	0.00023	0.51456	1.17
**V663**	-	0.063	4.702	1.0355	0.133	0.51216	0.00005	0.51170	1.64
**V670**	-	0.070	0.871	1.0818	0.752	0.51833	0.00033	0.51577	1.50
**V674**	1.1	0.064	1.558	0.7398	0.287	0.51364	0.00022	0.51267	1.24
**V679**	1.1	0.050	1.404	0.7338	0.316	0.51366	0.00009	0.51258	0.88
**V683**	3.0	0.051	5.186	0.5562	0.065	0.50970	0.00007	0.50948	3.29

* Age group as in Table S1

m measured isotope ratio

e isotope ratio corrected for the Venetia kimberlite eruption age (520 Ma)

References

1 Richardson, S. H., Poml, P. F., Shirey, S. B. & Harris, J. W. Age and origin of peridotitic diamonds from Venetia, Limpopo Belt, Kaapvaal-Zimbabwe craton. *Lithos* **112**, 785-792, (2009).

2 Stachel, T. *et al.* The trace element composition of silicate inclusions in diamonds: a review. *Lithos* **77**, 1-19, (2004).
